# Task Scheduling of Multiple Humanoid Robot Manipulators by Using Symbolic Control

**DOI:** 10.3390/biomimetics10060346

**Published:** 2025-05-24

**Authors:** Mete Özbaltan, Nihan Özbaltan, Hazal Su Bıçakcı Yeşilkaya, Murat Demir, Cihat Şeker, Merve Yıldırım

**Affiliations:** 1 Department of Electrical and Electronics Engineering, Faculty of Engineering and Architecture, İzmir Bakırçay University, 35665 İzmir, Türkiye; hazalsu.bicakci@bakircay.edu.tr (H.S.B.Y.); murat.demir@bakircay.edu.tr (M.D.); cihat.seker@bakircay.edu.tr (C.Ş.); 2 Department of Computer Engineering, Faculty of Engineering and Architecture, İzmir Bakırçay University, 35665 İzmir, Türkiye; nihan.ozbaltan@bakircay.edu.tr; 3 Department of Software Engineering, Faculty of Engineering, Karadeniz Technical University, 61080 Trabzon, Türkiye; merveyildirim@ktu.edu.tr

**Keywords:** humanoid robot manipulators, task scheduling, multiple degrees of freedom, inverse kinematics, ANN, optimization algorithms, symbolic discrete controller synthesis

## Abstract

Task scheduling for multiple humanoid robot manipulators in industrial and collaborative settings remains a significant challenge due to the complexity of coordination, resource sharing, and real-time decision-making. In this study, we propose a framework for modeling task scheduling for multiple humanoid robot manipulators by using the symbolic discrete controller synthesis technique. We encode the task scheduling problem as discrete events using parallel synchronous dataflow equations and apply our synthesis algorithms to manage the task scheduling of multiple humanoid robots via the resulting controller. The control objectives encompass the fundamental behaviors of the system, strict rules, and mutual exclusions over shared resources, categorized as the safety property, whereas the optimization objectives are directed toward maximizing the throughput of robot-processed products with optimal efficiency. The humanoid robots considered in this study consist of two pairs of six-degree-of-freedom (6-DOF) robot manipulators, and the inverse kinematics problem of the 6-DOF arms is addressed using metaheuristic approaches inspired by biomimetic principles. Our approach is experimentally validated, and the results demonstrate high accuracy and performance compared to other approaches reported in the literature. Our approach achieved an average efficiency improvement of 40% in 70-robot systems, 20% in 30-robot systems, and 10% in 10-robot systems in terms of production throughput compared to systems without a controller.

## 1. Introduction

Humanoid robots are programmed to mimic the actions and movements of humans, making them eligible for any kind of application, from industrial automation to personal assistance. They have sensors, actuators, and control units that enable them to perform complicated motions and act in response to their surroundings much like a human being.

Humanoid robots face several challenges that are frequently emphasized in the literature, including achieving stable locomotion and balance to operate effectively across diverse environments (e.g., walking on various surfaces), ensuring effective human–robot interaction for performing human-friendly tasks, demonstrating robustness and adaptability to execute complex movements such as crab walk and possessing cognitive capabilities for decision-making, object recognition, and responding to given commands.

To address these challenges, the humanoid robot design simulation is usually performed prior to physically creating the robot. Task scheduling is one of the most important issues in humanoid robot design. Task scheduling is the method of assigning and coordinating tasks between human and robot operators to realize maximum productivity while ensuring seamless collaboration. In the literature, task scheduling solutions have been predominantly explored through heuristic approaches, metaheuristic (MH) techniques, exact algorithms, simulations, and hybrid methodologies. For instance, Pereira et al. [1] present an algorithm using the GRASP MH for task scheduling and assignment in human–robot collaborative settings to demonstrate its effectiveness in productivity and flexibility improvement. Similarly, Pupa et al. [2] present a resilient online task scheduling framework that adapts to deviation and error in real time during execution to facilitate efficient human–robot collaboration.

Task scheduling in multiple humanoid robots to improve operational efficiency has significantly high practical benefits in real-world applications. By optimizing task allocation and coordination, industries such as manufacturing, healthcare, and services can achieve enhanced security and precision. For example, in the healthcare sector, humanoid robots can assist with patient mobility or provide surgical assistance, enabling them to be utilized in critical tasks with high precision and reliability.

Our research idea is derived from a comprehensive literature review and aims to address the challenges and problems outlined above. In this context, our research question is the following: How can task scheduling be optimized to enhance the overall performance and efficiency of collaborative robots?

In the literature, such problems are typically addressed either using classical control methods, which often suffer from high computational complexity, or metaheuristic approaches, which may introduce inaccuracies. In contrast, we observed that the task scheduling problem for multiple humanoid robots can be modeled as a feedback control problem. Accordingly, we propose a symbolic discrete controller synthesis approach that offers significantly better computational performance compared to classical control methods. Furthermore, unlike metaheuristic techniques, our method ensures the synthesis of a controller with formally verified correctness, thereby achieving high accuracy in task scheduling.

Inverse kinematics (IK) is another highly significant area of humanoid robot design that deals with determining the joint angles required to achieve a desired robot end-effector position. Optimal solutions to the IK problem are vital to guarantee correct and smooth motion of the robots. O’Flaherty et al. [3] provide thorough forward and IK derivation for the HUBO2+ humanoid robot in the context of being able to control a 27 degree-of-freedom (DOF) robot. Moreover, Ali et al. [4] introduce a closed-form IK joint solution for humanoids, presenting a generalized and efficient method which can be applied to various humanoid platforms. Said et al. [5] obtain an explicit, omnidirectional, analytical, and decoupled closed-form solution for lower limb kinematics of humanoid robot NAO. Their solution separates position and orientation analysis from the total Denavit–Hartenberg (DH) transformation matrices, thereby allowing efficient computation of joint angles.

The 6-DOF robotic arm consists of six serial links, making it capable of operating at any angle or along any trajectory. With computer technology developing at a high rate, the control of 6-DOF robotic arms is increasingly becoming challenging. Current control methods feature sophisticated algorithms to effectively address both inherent errors of the robotic arm and external disturbances. MH approaches to 6-DOF robotic arm control and optimization involve the utilization of sophisticated optimization algorithms to enhance the performance and precision of such robotic arms. Such approaches are very effective in addressing complex problems such as trajectory planning, error correction, and compensation for external disturbances. To minimize errors and improve stability, a fractional-order PID (Proportional Integral Derivative) controller [6], computer vision-based calibration framework, quasi-physical model, frequency response function, deep reinforcement learning methods, and gray wolf optimization algorithm (GWO) were utilized on 6-DOF robotic arms.

The use of control algorithms for robotic manipulators through simulation programs is a common topic studied by researchers. A neural network solver for kinematic control systems has been developed on the V-REP simulation platform [7]. In [8]; the focus is on the implementation of PID controllers in the MATLAB Simscape environment, and in [6] the MATLAB Simulink environment has been used. In this paper, the discrete controller synthesis technique has been used in simulation platforms such as MATLAB, Python, and Reax and in physical environments. In addition, artificial neural networks have been used to solve inverse kinematics and task planning problems in this study. In the proposed model, the artificial neural network has been optimized with the gray wolf optimization algorithm and superior results have been obtained.

The DCS technique was developed by Ramadge and Wonham in 1989 and was asserted as a theoretical framework for formal language models [9]. In this framework, synthesized controllers were proposed for specific control purposes. This technique has subsequently been used in many modeling methods, such as automata, Petri nets, and finite-state machines. In this context, Balemi et al. [10] have adopted an input-—output viewpoint, representing systems as finite-state machines or automata. An alternative approach was introduced by Maraninchi et al. [11], who proposed a synchronous language framework grounded in automata theory. However, these methodologies have faced significant limitations, particularly due to the state explosion problem, which poses challenges to scalability and practical implementation. In response to these challenges, a symbolic methodology based on labeled transition systems was proposed in [12]. This marked the emergence of symbolic discrete control techniques, which initially focused exclusively on Boolean variables. Subsequent studies, including References [13,14], offered solutions grounded in the synthesis algorithm. Building upon this foundation, a symbolic framework capable of addressing infinite-state systems was later introduced in the study by Berthier et al. [15] using the ReaX environment.

The literature comprises a broad spectrum of DCS work. Some of the notables among them are the use of current-state and initial-state opacity [16], the use of current-state opacity verification over time-labeled Petri nets [17], the integration of reinforcement learning and supervised control theories with DCS for the control of train traffic [18], its use in dynamic timed automata [19], and automata-based DCS for reconfigurable systems [20].

In the literature concerning the application of DCS in humanoid robots, Ishimizu et al. [21] have employed this method for the control of drones, specifically for takeoff, landing, and battery management. Additionally, Li et al. [22] utilized the DCS approach to regulate the movements of the robot. Zudaire et al. [23] explored how controller synthesis can facilitate the transition from old mission objectives to new ones, as well as the architectural restructuring necessary to incorporate new software actuators and sensors when required. Ref. [24] has identified a significant gap in the existing literature concerning the application of task scheduling in robotic studies. In the study, Gueye et al. [25] employed a discrete control approach based on automata to manage unmanned vehicles. In their methodology, they developed a model by implementing the DCS technique using an FPGA and integrating a task scheduling layer. Ref. [26] used DCS and task scheduling methods together to ensure that no tasks can be scheduled on a faulty processor.

Our study tackles the IK problem by framing it as an optimization challenge. We employ ANNs to develop the IK model and use MH algorithms to optimize the network’s weights and biases. This hybrid ANN-MH approach significantly enhances both accuracy and generalizability, effectively addressing the limitations of traditional IK solvers. Moreover, we extend this approach to address the task scheduling problem for multiple humanoid robots operating within a closed-loop system that integrates pipeline and parallel processes. By treating the task scheduling problem as a feedback control issue using symbolic DCS, we encode uncontrolled system behaviors as parallel dataflow equations in synchronous programming languages and incorporate desired specifications as control objectives. These objectives include safety algorithms and optimization goals, ensuring optimal token planning in pipeline channels during task scheduling. The synthesized controller, validated in both physical and simulation environments, guarantees desired system behaviors. The motivation for our study arises from the identified gap in the literature regarding the application of task planning in robotic studies, as noted in [24,27]. Addressing this gap is essential for advancing the field of robotics, particularly in improving the efficiency and reliability of humanoid robots in complex operational environments.

Contributions: Our integrated methodology not only closes gaps in task scheduling and IK for humanoid robot-identified gaps but also provides considerable improvements in performance, reliability, and efficiency.

- Symbolic DCS for Task Scheduling: We employ symbolic DCS to manage task scheduling for humanoid robots, ensuring strict rules and mutual exclusions through safety algorithms and maximizing energy efficiency in the shortest time possible.
- Synchronous Language Modeling: Synchronous programming languages simulate humanoid robot systems as a task scheduler manager through generating a controller using DCS and implementing it in the robot system.
- IK with MH Algorithms: The IK of robot joints is addressed with metaheuristic algorithms for enhancing the performance of multiple-degree-freedom robot arm manipulators.
- ANN for IK Model Development: We develop the inverse kinematics model using ANN and couple it with MH algorithms to train the network weights and biases for achieving maximum accuracy and generalizability.
- Experimental Validation: Our method is experimentally validated with high accuracy and performance compared to other methods in the literature.

The remainder of the paper is organized as follows. The next subsection provides a comprehensive literature review. Section 2 provides an overview of the general technical background of humanoid robots. Section 3 elaborates on how the inverse kinematics problem is addressed and presents detailed models of task scheduling among humanoid robots. Section 4 reports the results of our experimental evaluations. At last, Section 5 concludes the paper and suggests directions for future work.

### 1.1. Related Work

#### 1.1.1. Task Scheduling

Numerous studies in the literature have explored diverse applications of robotics across a variety of domains, as demonstrated in works such as [28,29,30]. Building on the historical evolution of humanoid robots and the fundamental challenges in their development, recent research has increasingly focused on equipping these systems with intelligent control strategies and learning-based approaches. These efforts are primarily driven by the growing need to cope with the complexity of tasks performed in dynamic and unstructured environments [31,32,33]. The overall goal is to enhance humanoid robots with greater flexibility, robustness, and adaptability. Various control models have been explored in the literature to improve motion and task execution in robotic systems. Among them, two-degree-of-freedom (2-DOF) systems have gained popularity due to their simplicity and suitability for real-world applications such as helicopter dynamics. In particular, 2-DOF helicopter platforms provide a valuable benchmark for evaluating novel control strategies in nonlinear and coupled dynamics, especially in discrete-time environments [34].

Ref. [35] presents LoRaStat, a low-power, portable potentiostat integrated with LoRaWAN for wireless communication, optimized for long-term monitoring of ammonium, nitrate, and calcium levels in aqueous environments. Ref. [36] presents scheduling algorithms that optimize job scheduling to maximize weight for batteryless IoT devices, addressing energy constraints with polynomial-time solutions and NP-Hardness for general cases. Ref. [37] demonstrates that with proper MOCAP calibration, wearable sensors can accurately monitor knee range of motion in TKA patients, offering a reliable alternative to traditional methods. Ref. [38] proposes a robust two-layer distributed adaptive learning control strategy for multi-agent robotic manipulators, enabling trajectory tracking and learning of nonlinear uncertain dynamics in a leader-follower framework.

#### 1.1.2. Inverse Kinematics Problem

In parallel with traditional control schemes, significant progress has been made in the field of estimating and tracking 6-DoF poses. However, many existing methods rely heavily on pre-defined CAD models or prior category knowledge, which limits their applicability to previously unseen objects. To overcome this limitation, BundleSDF is proposed as a co-designed method that integrates pose tracking with neural reconstruction. This model-free framework enables 3D reconstruction and causal 6-DoF tracking from monocular RGB-D sequences, even under challenging conditions such as occlusion, specular surfaces, or lack of texture [39]. Complementary to pose estimation, grasp generation in cluttered scenes has also received considerable attention. Contact-GraspNet addresses the limitations of previous approaches by providing an end-to-end network that predicts stable and diverse 6-DoF grasp poses directly from raw depth images. In particular, it avoids reliance on object labels or instance segmentation, which enhances its ability to generalize in unstructured environments [40]. To support these learning-based approaches, the accessibility and quality of the datasets play a crucial role. Existing 6-DoF pose estimation benchmarks often suffer from limited accessibility or outdated object models, reducing their utility in real-world robotic manipulation tasks. The HOPE dataset fills this gap by providing high-quality textured 3D models of 28 household objects, together with challenging and cluttered scene configurations, to serve as a robust benchmark for pose estimation tasks [41].

A major challenge that remains is the inverse kinematics problem in redundant systems, where the number of joints exceeds the number of degrees of freedom needed to control the end effector. This redundancy results in multiple feasible joint configurations for a single target pose. While this flexibility has clear advantages, including improved obstacle avoidance, fault tolerance, and configuration flexibility, it also complicates the learning process. Traditional multi-solution IK approaches address this by partitioning the joint space into sub-regions, solving for each separately and then merging the results [42,43,44]. In this study, metaheuristic optimization algorithms are used to efficiently explore the high-dimensional joint space and determine optimal configurations for the desired end-effector objectives.

#### 1.1.3. Symbolic Controller Synthesis

In terms of control-level improvements, the integration of advanced control strategies, intelligent optimization techniques, and accurate kinematic modeling has significantly enhanced the performance of humanoid robot manipulators. Three core research areas stand out: discrete controller synthesis (DCS), inverse kinematics (IK), and artificial neural network (ANN)-based optimization. DCS ensures the safe and deterministic coordination of robot actions [14,45], while solving the IK problem is essential for achieving accurate end-effector positioning [44], especially in systems with redundant degrees of freedom [42]. In addition, the combination of metaheuristic optimization techniques with ANN models has proved effective in dealing with the nonlinearities and real-time constraints commonly encountered in complex robotic systems. Recent developments in the synthesis of discrete-event controllers highlight the growing interest in combining formal verification techniques with data-driven learning. For example, the DEEPDECS framework proposed by Calinescu et al. enables correct-by-construction controllers for autonomous systems by integrating deep neural networks (DNNs) for perception [46]. Similarly, symbolic controller synthesis techniques have been applied to nonlinear and underactuated systems such as quadcopters, providing reliable path planning and control without the need for additional hardware [24,47,48,49,50]. These methods have shown superior performance compared to traditional fractional order PID (FOPID) controllers in both dynamic behavior control and trajectory generation tasks.

## 2. Technical Background of the Common Problems Encountered in the Task Scheduling of Humanoid Robots

Humanoid robots are systems that have a human-like physical structure and are designed to perform specific tasks. They are characterized by their ability to perform a given system task using their limbs. They are also capable of interacting with the environment and with each other and have a degree of autonomy. Accordingly, their interactions, both with the external environment and with other humanoid agents, and their task-planning processes must be managed in accordance with desired control objectives.

Humanoid robots usually consist of several robot manipulators with a high degree of freedom working in a coordinated manner. To enable controlled movement from one coordinate to another, the inverse kinematics (IK) problem must first be solved as a fundamental step in motion planning.

### 2.1. Six-Degree-of-Freedom Robot Manipulators

Humanoid robots are articulated manipulators operating with multiple degrees of freedom. One of their primary functions is to mimic the dexterity and flexibility of typical human limbs. In this study, a humanoid robot consisting of two 6-DOF manipulators working in a coordinated manner to perform complex tasks was used.

**Figure 1:**
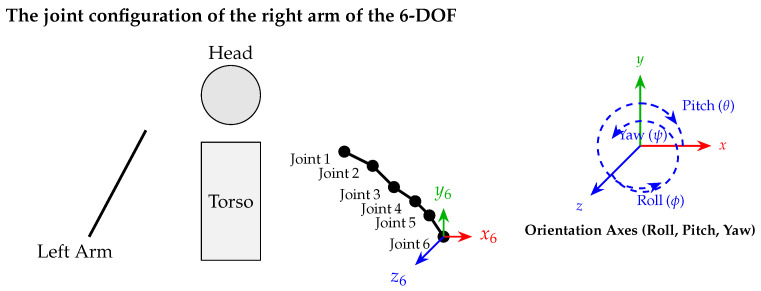
The joint configuration of the right arm of the 6-DOF humanoid robot and the roll, pitch, and yaw rotations of its end effector.

Six-degree-of-freedom humanoid robots can perform displacement motions such as sliding left/right on the X-axis, descending/ascending on the Y-axis, and moving forward/backward on the Z-axis. It can also perform rotational motions such as roll (ϕ) around the X-axis (arm rotation around itself), pitch (θ) around the Y-axis (tilting up and down) and yaw (ψ) around the Z-axis (left/right rotation). All movements of the robot are presented in  Figure 1.

In this paper, an inverse kinematics (IK) model is proposed for the arms of a 6-DOF humanoid robot using artificial neural networks. Metaheuristic algorithms are used to optimize the weights and biases of the artificial neural network. This results in lower error rates, higher accuracy rates, and better solutions without getting stuck in local minima.

### 2.2. Inverse Kinematics Problem

There are two basic problems that restrict the movements of 6-DOF humanoid robots. The first of these is the forward kinematics problem, where the position of the end effector is determined according to the given joint angles. The second is the inverse kinematics problem, where all the joint angles required to bring the end effector to the desired position are calculated.

In the inverse kinematics problem, which is also the subject of this study, the joint angles $\theta =[\theta_{1},\theta_{2},\ldots ,\theta_{n}]$ need to be calculated in order to bring the end effector to the desired position $P_{\mathrm{target}}$. Mathematically, the joint angles are calculated as follows:

(1) $$f\left(\theta \right)=P_{\mathrm{target}}$$

where f(θ) represents the forward kinematics function. Forward kinematics problems are solved using trigonometric or algebraic methods and have a single solution. However, inverse kinematics problems are solved using closed-form or iterative methods. They have more than one solution or no solution at all.

In order to reduce the complexity of traditional methods and produce faster solutions, the inverse kinematics problem is solved using artificial neural networks. The inputs of the network are the positions of the end effector of the 6-DOF humanoid robot in free space, and the outputs are the joint angles corresponding to these positions. The positions of the end effector corresponding to random joint angles are calculated using forward kinematics formulas. The artificial neural network is trained with the obtained data. The weights and biases of the network are optimized using metaheuristic algorithms. Metaheuristic algorithms increase both the accuracy rate of artificial neural networks and improve error metrics (such as MSE and $R^{2}$).

### 2.3. Artificial Neural Network (ANN) Model

**Figure 2:**
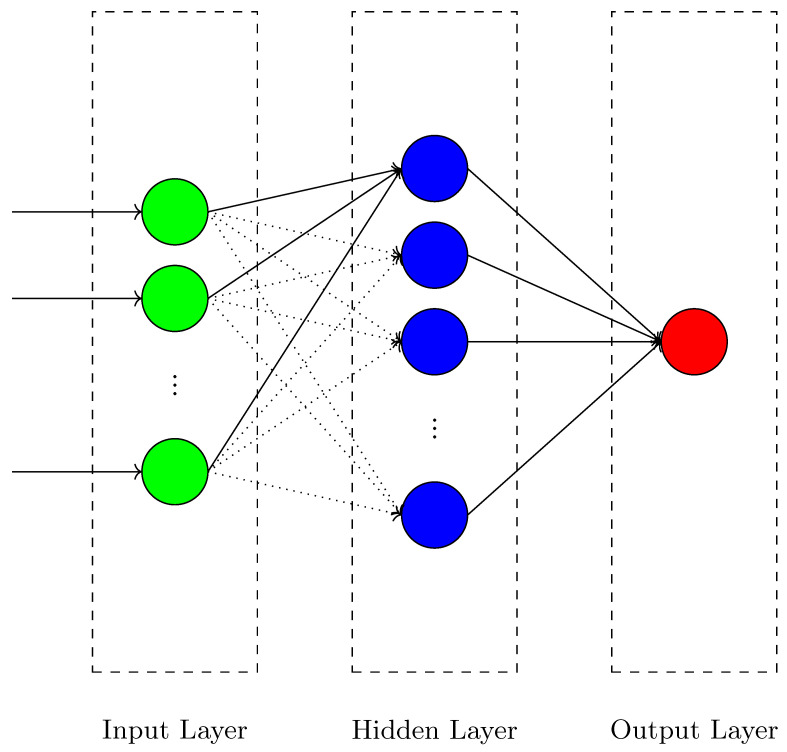
General artificial neural network (ANN) architecture.

The artificial neural network model developed within the scope of this study consists of three components. The input layer contains information about the position of the end effector of the 6-DOF humanoid robot. In the hidden layer, the input data are processed and inferences are made. The learning process takes place in this layer. The output layer contains the joint angles estimated by the network. The three-layer artificial neural network structure is given in Figure 2. The outputs of the network are calculated using a standard feedforward structure whose equation is given below.

(2) y=f(Wx+b)

Here, *W* is the weight matrix, *x* is the input vector (i.e., the position of the end effector), *b* is the bias term, and f(·) is the nonlinear activation function. The weights and biases of the network are optimized using metaheuristic algorithms. In this way, the optimum artificial neural network structure in terms of the number of layers and learning rate is created.

### 2.4. Metaheuristic Approaches

The metaheuristic algorithms utilized in this study are as follows.

Particle Swarm Optimization (PSO): It has been observed that the movements of birds and fish moving in flocks to find food affect other individuals in the flock, and the flock reaches its goal more easily. Inspired by this observation, it is an optimization algorithm developed in 1995 [51]. The best solution is achieved by changing the speeds and positions of the particles.

Ant Colony Optimization (ACO): This algorithm is inspired by mimicking the behaviors and interactions of individual ants in an ant colony [52]. Ants leave trails in their environment to reach food sources. These trails help other ants find the right route, while the trailing ants adjust their movements according to the density of the trails. The ant colony algorithm similarly creates a roadmap by searching for possible solutions and using a series of signs and trials to reach better solutions. Probabilistic decision-making mechanisms work while trying to reach the optimum solution.

Artificial Bee Colony (ABC) Algorithm: This algorithm models the foraging behavior of honey bees [53]. Bees tend to explore new food sources based on the information shared by other bees in order to find the most efficient source. When all known food sources are exhausted, the bees continue their search randomly in different locations.

Gray Wolf Optimization (GWO): This algorithm is based on the hunting model of a gray wolf pack [54]. There are four types of wolves, alpha, beta, delta, and omega, according to their role in the pack. The alpha holds the greatest ’authority’ in decision-making and leading the pack. Following the alpha are the beta and delta wolves, which are subordinate to the alpha yet dominant over other members. The omega wolf is always subordinate to the more dominant wolves in the hierarchy.

Coyote Optimization Algorithm (COA): It is a population-based algorithm classified both as a swarm intelligence and an evolutionary heuristic approach [55]. The algorithm is inspired by behaviors of coyotes. Unlike the GWO algorithm, the COA is based on a different algorithmic structure. The COA does not focus on social hierarchy and dominance rules. Instead of merely emphasizing hunting behavior, it also incorporates social structures and experience sharing among individuals.

All of the aforementioned metaheuristic algorithms were employed to optimize the weight and bias parameters of the ANN model. The obtained results were evaluated using the Mean Squared Error (MSE) method:

(3) $$\mathrm{MSE}=\frac{1}{n}\sum_{i=1}^{n}{\left(y_{i}-{\hat{y}}_{i}\right)}^{2}$$

where $y_{i}$ represents the actual joint angles, while ${\hat{y}}_{i}$ denotes the predicted joint angles.

In addition to MSE, the performance of each model was also evaluated using the coefficient of determination ($R^{2}$). This enabled a better approximation of the predicted outputs to the actual values. In the MSE method, the squared differences between the actual and predicted joint angles are summed and divided by the number of samples. When calculating $R^{2}$, the ratio of the residual sum of squares to the total sum of squares is computed and then subtracted from 1. A lower MSE value and an $R^{2}$ value closer to 1 indicate the accuracy and generalizability of the proposed inverse kinematics model. These evaluation metrics provide a comprehensive understanding of the predictive performance of ANN models optimized using different metaheuristic algorithms.

## 3. Task Scheduling of Multiple Humanoid Robot Manipulators by Using Symbolic Control

**Figure 3:**
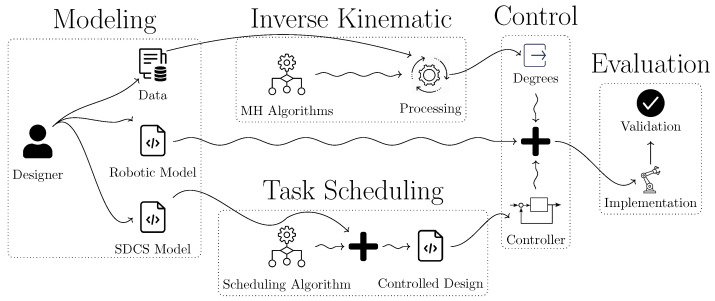
Workflow of our approach for task scheduling of multiple humanoid robot manipulators.

This study addresses task scheduling for multiple humanoid robots through the use of the symbolic discrete controller synthesis technique. The workflow of our study is presented in  Figure 3. As illustrated in the figure, two distinct problems (inverse kinematics and task scheduling) commonly encountered in robot design are addressed separately, and solutions developed for these two problems are then integrated. Our study is structured into a five-phase modeling framework, as shown in the figure.

In the first phase, the system behaviors that have not yet been controlled are modeled both as a discrete controller synthesis problem and within a virtual robotic environment. Additionally, a database is created for the joint angles and target positions of the robots.

The system specifications (control objectives) are then associated with our plant model, and through the integration of the developed scheduling algorithms, a controller is generated using our safety and optimization synthesis algorithms. This controller is subsequently implemented within the virtual robotic environment for task scheduling operations. Furthermore, the inverse kinematics problem in the robots is addressed by utilizing the database, and the degrees of the joints are determined using the developed MH algorithms to achieve the desired position. This process is also integrated into the virtual robotic environment. The results obtained from the simulation environment are validated in a physical setting.

The modeling framework presented below is systematically detailed, providing an in-depth overview of the approach.

### 3.1. Overview

This study focuses on optimizing task scheduling for multiple humanoid robots operating within a closed-loop system as part of a pipeline. In this context, the inverse kinematics problem for each limb of the humanoid robots is addressed as the first step.

In the designs we considered in this work, the inverse kinematics problem is addressed using 6-DOF robot manipulators as an example for the limbs of humanoid robots. Initially, a database is created for the joint angles and target positions of the 6-DOF robot manipulators. Subsequently, various metaheuristic approaches and hybrid models commonly used in the literature are employed to complete the training process for our database. Finally, during the implementation phase in both physical and simulation environments, the trained models are used to generate output angles, ensuring that the humanoid robots can optimally achieve the necessary joint angles to move from one position to another.

In this study, humanoid robots are considered as two-pair 6-DOF manipulators, and the task scheduling problem for multiple humanoid robots operating within a closed-loop system that combines pipeline and parallel processes is addressed. The inverse kinematics problem for each limb of each humanoid robot, as discussed in the previous context, is solved using metaheuristic approaches. The task scheduling problem of multiple humanoid robots, on the other hand, is treated as a feedback control problem using the symbolic discrete controller synthesis technique. Initially, the uncontrolled system behaviors for the humanoid robots are encoded as parallel dataflow equations in synchronous programming languages. Subsequently, the desired specifications are incorporated into the model as control objectives. These control objectives fall into two categories: the first includes safety algorithms, such as strict rules and mutual exclusions, while the second focuses on optimization objectives, which are used to plan the tokens in the channels of the pipeline in the most optimal manner during task scheduling. Once the model is complete, during the compilation process, the controller, which always ensures the desired system behaviors, is synthesized by applying the safety and optimization algorithms. Finally, this synthesized controller is integrated into the physical or simulation environment to be validated.

### 3.2. Inverse Kinematics Model of 6-DOF Robot Manipulators

**Figure 4:**
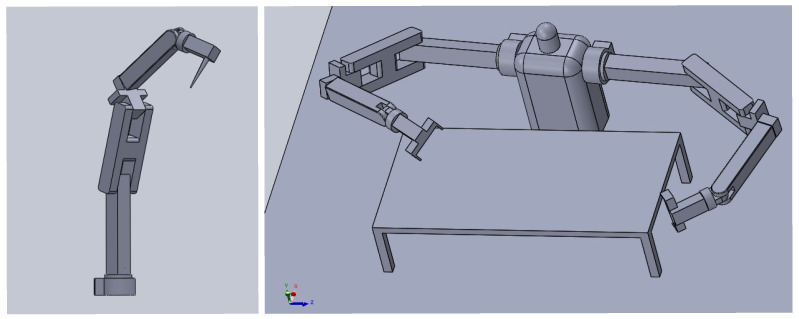
General view of the 6-DOF manipulator and humanoid robot.

Within the scope of this study, the humanoid robots are considered as two pairs of 6-DOF manipulators. The general view of the 6-DOF manipulator and humanoid robot is shown in  Figure 4. In this context, the kinematic problem in humanoid robots is also addressed through 6-DOF manipulators.

The mathematical model of the 6-DOF manipulator is expressed as follows. The homogeneous transformation matrix given in Equation (10) is obtained using all transformation matrices given in Equations (4) to (9). $R_{k}$ and $T_{k}$ represent the rotation matrix and the translation matrix of joint *k*, respectively.

(4) $$R_{x}=\left[\begin{array}{cccc} 1 & 0 & 0 & 0\\ 0 & \cos\alpha & -\sin\alpha & 0\\ 0 & \sin\alpha & \cos\alpha & 0\\ 0 & 0 & 0 & 1 \end{array}\right]$$

(5) $$T_{x}=\left[\begin{array}{cccc} 1 & 0 & 0 & a\\ 0 & 1 & 0 & 0\\ 0 & 0 & 1 & 0\\ 0 & 0 & 0 & 1 \end{array}\right]$$

(6) $$R_{y}=\left[\begin{array}{cccc} \cos\beta & 0 & \sin\beta & 0\\ 0 & 1 & 0 & 0\\ -\sin\beta & 0 & \cos\beta & 0\\ 0 & 0 & 0 & 1 \end{array}\right]$$

(7) $$T_{y}=\left[\begin{array}{cccc} 1 & 0 & 0 & 0\\ 0 & 1 & 0 & b\\ 0 & 0 & 1 & 0\\ 0 & 0 & 0 & 1 \end{array}\right]$$

(8) $$R_{z}=\left[\begin{array}{cccc} \cos\gamma & -\sin\gamma & 0 & 0\\ \sin\gamma & \cos\gamma & 0 & 0\\ 0 & 0 & 1 & 0\\ 0 & 0 & 0 & 1 \end{array}\right]$$

(9) $$T_{z}=\left[\begin{array}{cccc} 1 & 0 & 0 & 0\\ 0 & 1 & 0 & 0\\ 0 & 0 & 1 & c\\ 0 & 0 & 0 & 1 \end{array}\right]$$

(10) $$T=\prod_{k=1}^{n}R_{k}T_{k}$$

**Figure 5:**
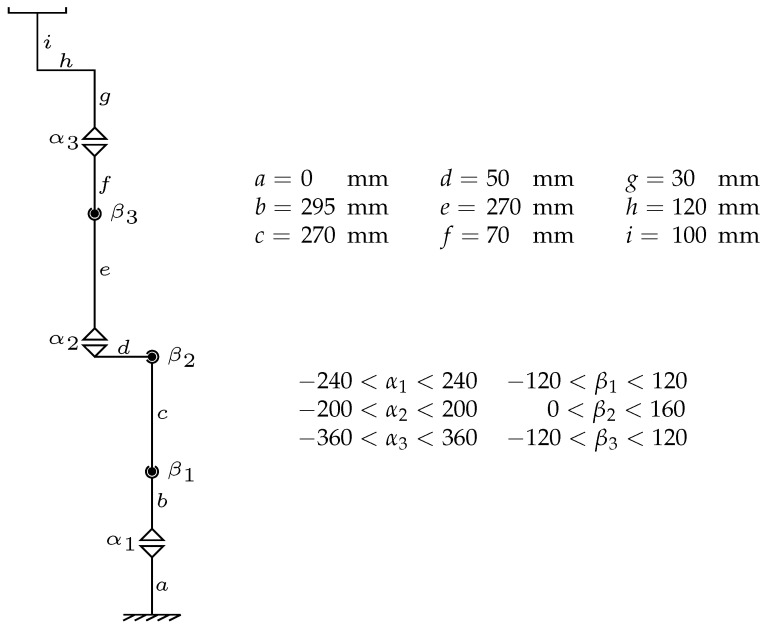
Kinematic scheme, link lengths, and joint angle limits of the 6-DOF manipulator.

The physical 6-DOF manipulator used in this study, along with its kinematic scheme when all joint angles are set to zero, link lengths, and joint angle limits, is depicted in  Figure 5.

In this study, various optimization algorithms are employed to solve the inverse kinematics of 6-DOF manipulators. A multilayer artificial neural network (ANN) model is used, with artificial neurons positioned between the input and output layers, and a single hidden layer containing 10 neurons. Additionally, optimization processes involve evolutionary algorithms such as Particle Swarm Optimization (PSO), which is based on social interactions between particles; Ant Colony Optimization (ACO), which uses pheromone-based communication among artificial ants; Artificial Bee Colony (ABC), inspired by the behavior of honeybees searching for nectar; Gray Wolf Optimization (GWO), based on the hunting behavior of wolves; and the Coyote Optimization Algorithm (COA), which mimics the social structure and hunting methods of coyotes. These algorithms are effectively applied to solve the inverse kinematics problem of 6-DOF robot manipulators for finding optimal solutions.

Swarm intelligence algorithms are highly effective in controlling the behavior of robotic systems, particularly for avoiding local minima during complex optimization tasks. In the literature, algorithms such as PSO, ACO, ABC, GWO, and COA have demonstrated strong performance in solving inverse kinematics problems by providing robust and accurate solutions. In this study, optimization was performed using these algorithms, where the joint angles generated through inverse kinematics were validated by comparing the corresponding end-effector positions obtained via forward kinematics. This approach ensures both the accuracy and reliability of the proposed kinematic solutions.

### 3.3. Symbolic Model of Humanoid Robot Manipulators

**Figure 6:**
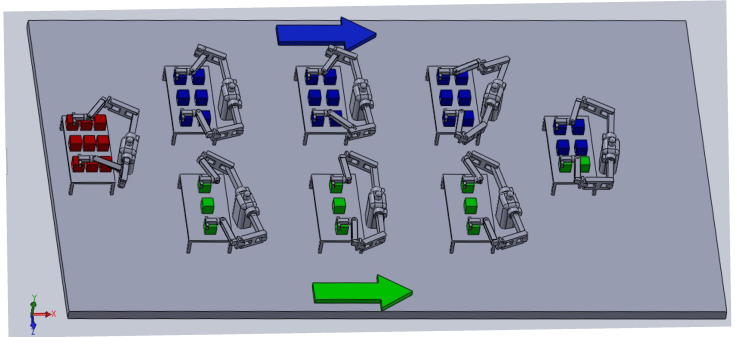
An illustrative task scheduling scenario in multiple humanoid robots.

Task scheduling optimization in humanoid robots has been modeled as a symbolic discrete controller synthesis problem. The system design, as illustrated in  Figure 6, considers robots arranged in both pipeline and parallel configurations, where the workflow proceeds through two separate sub-lines. The final robot in the sequence processes tokens arriving from both lines. However, to ensure optimal performance, the tokens arriving from both branches must be processed in a balanced manner, requiring synchronization between the execution times of the robots along each line. This necessitates a cost-efficient optimization procedure. In line with this objective, the workflow scenarios as depicted in the figure are systematically modeled, and discrete controller synthesis is carried out in detail, as presented below.

#### 3.3.1. Activation Model

The activation model is designed to control whether each robot in a closed system is actively performing its assigned task. This model is formulated as a parallel synchronous dataflow equation, as shown below:

(11) $$R_{i}=\mathrm{if}\;c_{i}\;\mathrm{then}\;A_{a}\;\mathrm{else}\;A_{p},\;\forall i\in \left\{1,2,\ldots ,|R|\right\},$$

where R denotes the set of robots within the system, and |R| represents the total number of robots. The variable *c* is a controllable input parameter, where each robot is associated with an individual control input $c_{i}$. The domain A is a custom-defined Boolean-like domain with two discrete values, a,p, where $A_{a}$ indicates that a robot is in an active state, and $A_{p}$ indicates that the robot is in a passive state.

#### 3.3.2. Mutual Exclusion Constraints

The grammar that can be used to formalize the constraints related to the interactions between collaborative robots working on shared resources, as well as their environmental relations, is formulated as follows: (12)
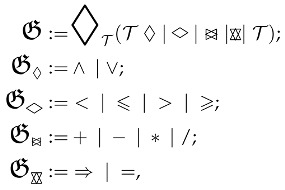

where the grammar represented by G illustrates the formalization of the system; the symbol := denotes the definition of the corresponding grammar; T represents any transition defined within the system model, such as transitioning a robot into an active state.

#### 3.3.3. Token Model

The tokens exchanged between humanoid robots operating on a pipeline are formalized in the following dataflow equation:

(13) $$\begin{array}{cc} P_{i}= & \;\left(R_{i}=A_{a}\right)\wedge e_{i}; \end{array}$$

(14) $$\begin{array}{cc} D_{i}= & \;\left(R_{ij}=A_{a}\right)\wedge e_{j}, \end{array}$$

where $P_{i}$ represents the tokens processed by robot *i*; $D_{i}$ denotes the delivered tokens of robot *i*; $R_{ij}$ represents the robot *j* that delivers to robot *i*; and *e* indicates the completion of a process on a token, after which it is fired.

**Figure 7:**
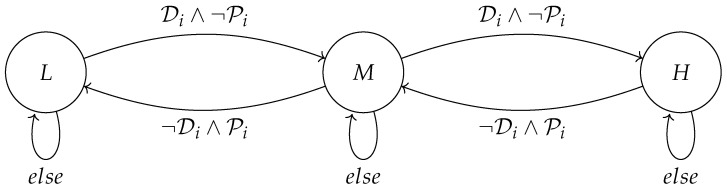
Token model transition between humanoid robots.

The tokens processed by humanoid robots exist in an infinite domain. However, for higher-performance coding, the tokens are abstracted in a finite domain, as illustrated in  Figure 7.

To provide a more detailed explanation of our task scheduling algorithm, the symbolic discrete controller synthesis approach begins by modeling the uncontrolled system behaviors and the desired specifications as parallel dataflow equations. This modeling captures strict rules governing robot behaviors, including conditions such as mutual exclusions, as well as optimization objectives within the system.

#### 3.3.4. Control

After modeling the system behaviors and desired specifications, controller synthesis is performed through the application of our safety and optimization synthesis algorithms.

In this context, our safety objectives such as the activation model and mutual exclusion constraints are formulated as follows:

(15) $$S_{glob.}=⋀S_{i},$$

where $S_{i}$ represents all the safety objectives in our desist model. $S_{glob.}$, on the other hand, ensures that evaluation is performed on controllable variables while remaining at a constant value of 1, acting as an invariant.

With the cost function formulated as shown below, the robots are coded to consume the maximum number of tokens with the optimal cumulative cost through our optimization algorithm:

(16) $$O=\sum_{i\in |R|}R_{i}⊥\left\{L,M,H\right\},$$

where ⊥ represents the quantity of tokens present in the channels between the humanoid robots.

#### 3.3.5. Integration

Finally, after the controller is synthesized, it is translated into languages such as C and Verilog using our software tools like SDCS2C and SDCS2HDL. This translation enables integration into environments such as Python, MATLAB, and VREP or into physical microcontrollers. The overall workflow of the study is outlined in Algorithm 1.
**Algorithm 1 ** Task Scheduling of Multiple Humanoid Robot Manipulators by Using Symbolic Control1:**Stage 1: Inverse Kinematics Dataset Generation**2:**for** each sample j=1…N **do**3:     Generate random joint angles: $\theta^{\left(j\right)}∼U\left(\left[\theta_{i,\min},\theta_{i,\max}\right]\right)$4:     Compute end-effector position: $x^{\left(j\right)}=f\left(\theta^{\left(j\right)}\right)$5:     Add to dataset: $D\leftarrow D\cup \{\left(x^{\left(j\right)},\theta^{\left(j\right)}\right)\}$6:**end for**7:** **8:**Stage 2: Neural Network Training (via GWO)**9:Initialize weights: ϕ∼random10:**while** termination criterion not met **do**11:     Update weights using Grey Wolf Optimizer:12:     $\phi \leftarrow \phi -\eta \;\cdot \;\nabla_{\phi }L\left(\phi \right)$13:**end while**14:** **15:**Stage 3: Task Definition and Scheduling**16:**for** each task $T_{i}$ **do**17:     Determine activity: $A_{i}\left(t\right)\in \left\{0,1\right\}$18:     Update task queue: $\delta_{i}\left(t+1\right)=\delta_{i}\left(t\right)+A_{i}\left(t\right)-C_{i}\left(t\right)$19:     Enforce mutual exclusion: $A_{i}\left(t\right)\cdot A_{j}\left(t\right)=0,\;\forall i\neq j$20:**end for**21:** **22:**Stage 4: Controller Synthesis**23:Objective: $\min\sum_{i,t}w_{i}\;\cdot \;\delta_{i}\left(t\right)$24:Subject to: $\sum A_{i}\left(t\right)\leq k$25:Generate control policy: u(t)=π(δ(t),A(t))26:** **27:**Stage 5: Simulation and Performance Comparison**28:Run simulations for both uncontrolled and controlled systems29:Compare metrics: task completion time, conflicts, regulation, queue length

## 4. Experimental Evaluation

The experimental work of this study is addressed in two stages. The dataset of 1,700,000 data points was generated by simulating various motion scenarios for a 6-DOF robot manipulator, with joint angles ranging from −50 to +50 degrees. The data were collected by systematically varying joint angles and recording corresponding end-effector positions, which were then used to train the inverse kinematics model. For each configuration, the corresponding PSO, ACO, ABC, GWO, and COA were calculated using forward kinematics. The dataset was then created by pairing the generated joint angles with their respective end-effector positions, ensuring a comprehensive representation of the manipulator’s kinematic behavior. The results were then validated in both MATLAB Simulink and a physical setup. The second stage of the study, which involves the task scheduling problem of multiple humanoid robots, was encoded in the discrete controller synthesis environment, ReaX, through its own synchronous programming language. For validation of the experimental phase, system behaviors modeled in both Python (version 3.8.5) and MATLAB (version 2021a) Simulink environments were integrated with the controller synthesized in the ReaX environment, and the results are presented below.

Swarm algorithms are crucial for avoiding local minima in robot motion control. According to a comprehensive analysis in the literature, algorithms such as PSO, ACO, ABC, GWO, and COA have emerged as particularly effective in this regard. The performance evaluation of different optimization algorithms in modeling joint angles ($\alpha_{1}$, $\alpha_{2}$, $\alpha_{3}$, $\beta_{1}$, $\beta_{2}$, and $\beta_{3}$) using an ANN for the inverse kinematics problem of a 6-DOF robot manipulator is presented in Table 1. The weights and biases of a single hidden layer comprising 10 neurons were designated as search parameters. The experiments were conducted using MATLAB, with the number of iterations and the population size for the optimization algorithms set to 100 and 1000. According to the table, the performance of the models is evaluated based on the proximity of the MSE value to zero and the coefficient of determination ($R^{2}$) value to one. The GWO algorithm achieved the lowest MSE value, surpassing other algorithms by 470%, 25,133%, 26,737%, and 8912% in terms of the MSE criterion. Similarly, GWO demonstrated superior performance by reducing the MSE by 25,780%, 30,395%, 5741%, and 35,011% compared to alternative methods. Furthermore, GWO attained the lowest MSE value, outperforming competing algorithms by 13,842%, 3383%, 179%, and 2460%. The algorithm also exhibited the lowest MSE value, exceeding the performance of other methods by 5233%, 4913%, 4067%, and 9476%. Additionally, GWO outperformed other algorithms by 465%, 450%, 462%, and 218% in terms of MSE reduction. Lastly, GWO achieved the lowest MSE value, surpassing other methods by 560%, 552%, 1196%, and 576%.

Overall, GWO demonstrated the highest efficacy in optimizing all ANN models presented in this study. Nevertheless, several strategies can be explored to further enhance modeling accuracy. Future research could focus on experimenting with novel algorithms, adjusting population sizes, and integrating advanced metaheuristic approaches into ANN models for the inverse kinematics solution of robotic manipulators. The mean performance of the GWO results was considered, and a comparative analysis with similar studies based on Taguchi Optimization, the Sequential Mutation Genetic Algorithm (SMGA), and Adaptive Particle Swarm Optimization (APSO) is provided in Table 2.

**Figure 8:**
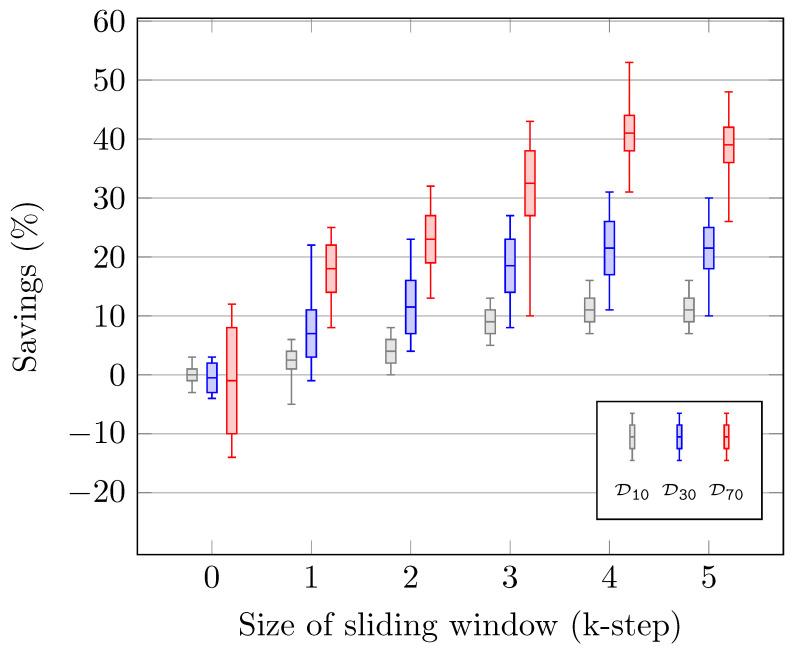
Comparison of task scheduling performance results.

Figure 8 presents a comparative performance analysis of our task scheduling algorithm. The colored bars in the figure represent designs containing varying numbers of humanoid robots. Specifically, the designs labeled $D_{10}$, $D_{30}$, and $D_{70}$ correspond to configurations with 10, 30, and 70 humanoid robots, respectively, each performing an activity with both sequential and parallel processes. This task scheduling comparative performance analysis has been implemented in a Python environment, with 100 trials conducted for each design, and the results are displayed in the bars. The lower and upper whiskers indicate the outlier points in the experiments, while the mean is represented by the median. The other segments are denoted by the lower and upper quartiles. On the x-axis, the k-step value of our optimization algorithm is represented, while the performance criterion is expressed as a percentage on the y-axis.

In the conducted experiments, our symbolic discrete controller synthesis approach was evaluated in terms of task scheduling performance on multiple humanoid robots. Within the scope of the experiments, when the designs are evaluated using dataflow-based modeling formalisms, each humanoid robot is represented as an actor (node), while the edges (arcs) correspond to the interactions between the robots. The objective of performance improvement in this context is to maximize the number of tokens fired by the final actor (robot), which serves as an indicator of overall system efficiency.

The same experimental environment was utilized under two conditions: with and without the integration of the SDCS-based controller. A value of *k*-step =0 indicates that no optimization was applied. The 0% point on the *y*-axis represents the case in which the controller was not integrated into the system. As expected, when input values were assigned randomly, approximately 50% of the cases demonstrated a performance improvement, while the remaining 50% showed a decrease. However, with the application of the optimization algorithm, the average performance consistently improved as the *k*-step value increased. After *k*-step =4, a plateau in performance gain was observed, suggesting that further increases in *k*-step no longer contributed to significant improvements. This indicates that the effect of future-oriented optimization had reached its limit or become negligible. Finally, the observed increase in bar height with a higher number of robots in the design is attributed to the growing number of combinations resulting from the randomness in the experimental outcomes.

The computational tool performance is presented in Table 3, which details both the computational time and the maximum memory usage as a function of the number of robots within a closed system. The table demonstrates the application of cumulative sum optimization over a time window of k-steps. As the value of k increases, signifying an extended lookahead within the time window, the computational time also increases. However, this rise in computational time is accompanied by a corresponding increase in the reliability of the optimization results. Notably, this increase in the time domain is scalable. As seen in the table, the computational time increases in proportion to the number of robots in the system. It is crucial to note, however, that the compilation process is carried out only once. Following the initial compilation, the controller operates in real time on the supervisory system.

Our proposed approach is compiled once after modeling and then operates dynamically in real time. However, when changes need to be made to the system, the model must be updated, and in such cases, the compilation process needs to be repeated. After this update, the system continues to operate in real time.

The results obtained through the experimental evaluation have been thoroughly reported and validated both in simulation and in a physical environment. The symbolic discrete control synthesis technique we employed functions as a model checking tool, thereby ensuring formal verification [15].

In this study, energy consumption is not directly addressed; however, energy efficiency has been indirectly improved as a result of faster and more coordinated task execution. In the literature, the task scheduling problem in robotic systems has been addressed in [59,60,61] using classical control methods or heuristic approaches. Ref. [62] introduces ISC-QL, a novel edge server placement algorithm for Intelligent Transportation Systems (ITS) that integrates Improved Spectral Clustering and Q-Learning. The method first clusters base stations by location and user density, then refines server placement using reinforcement learning. Experimental results using real-world data show that ISC-QL significantly enhances load balancing, reduces energy consumption, and lowers communication delay. However, our approach demonstrates significantly higher performance and a level of formal accuracy compared to the results of these works. Our method not only tackles the task scheduling problem in robotic systems with high performance and formal precision but also presents a novel integration with the inverse kinematics problem. Thus, our pioneering work presented in this paper would be for future research in this area.

## 5. Conclusions and Future Work

In this paper, we propose a modeling framework for task scheduling in multiple humanoid robot manipulators using the symbolic discrete controller synthesis technique. Our models have been systematically encoded in the ReaX environment by means of the ReaX’s parallel synchronous language. By incorporating control objectives into the model, the safety and optimization algorithms we applied effectively manage task scheduling operations, and our approach successfully achieves satisfying the desired control objectives. Additionally, the inverse kinematics problem for robots consisting of two pairs of 6-DOF manipulators has been trained using metaheuristic methods to enable movement from one position to another. Our approach, addressing two distinct key topics, has been integrated within the framework and the results obtained in the experimental evaluation environment are reported in detail. The results demonstrate that our approach outperforms those found in the literature, and the experimental evaluations have successfully validated our method.

In real-world applications, limitations in task scheduling often stem from scalability issues, particularly when deployed in large-scale or distributed systems. To address these challenges, future work will explore the integration of our proposed method with advanced machine learning techniques, such as reinforcement learning or neural architecture search, which can enable adaptive decision-making and improved generalization across diverse environments. Moreover, the inverse kinematics problem, as approached with metaheuristic algorithms in this study, may lead to approximate or suboptimal solutions. In safety-critical applications, such as robotics in healthcare or aerospace systems, ensuring correctness and reliability is essential. Therefore, future research will also focus on combining metaheuristic approaches with formal verification techniques or constraint-based solvers to ensure both flexibility and correctness. Additionally, we plan to investigate the real-time performance and hardware deployment of the proposed framework to assess its practical viability in industrial settings.

The modeling approach we propose can be easily applied to similar systems with different control objectives in future work. The optimization control algorithms we have used are pessimistic, and new approaches can be developed with the implementation of novel control algorithms. The metaheuristic algorithms used for inverse kinematics within the scope of this study can be hybridized with other optimization algorithms to achieve higher accuracy results. Alternatively, the reliability of results obtained through advanced machine learning algorithms, such as deep reinforcement learning, can be tested. To enable dynamic task scheduling in unstructured environments, future work may explore the integration of deep reinforcement learning algorithms into the proposed framework. Such methods could enhance adaptability and decision-making in complex, real-time multi-robot scenarios. Finally, our work can be extended to effectively operate in dynamic and unstructured environments that require real-time adaptation.

## Figures and Tables

**Table 1:** Performance evaluation of different optimization algorithms in modeling joint angles using ANN.

		Joint Angles					
Method	Error	$\alpha_{1}$	$\alpha_{2}$	$\alpha_{3}$	$\beta_{1}$	$\beta_{2}$	$\beta_{3}$
PSO [51]	MSE	0.0003	0.0359	0.0140	0.0229	0.0135	0.0284
	$R^{2}$	0.9992	0.8819	0.9536	0.9235	0.9547	0.9063
ACO [52]	MSE	0.0141	0.0337	0.0138	0.0270	0.0033	0.0275
	$R^{2}$	0.9561	0.8889	0.9569	0.9118	0.9882	0.9097
ABC [53]	MSE	0.0150	0.0279	0.0299	0.0051	0.0002	0.0282
	$R^{2}$	0.9536	0.9082	0.9000	0.9825	0.9994	0.9073
GWO [54]	MSE	0.0001	0.0007	0.0025	0.0001	0.0001	0.0061
	$R^{2}$	0.9998	0.9976	0.9914	0.9998	0.9996	0.9805
COA [55]	MSE	0.0050	0.0650	0.0144	0.0311	0.0024	0.0133
	$R^{2}$	0.9831	0.7878	0.9541	0.9086	0.9914	0.9553

**Table 2:** Performance comparison of optimization methods for the inverse kinematics problem.

Method	Error		References
GWO	0.0016	(MSE)	Proposed Method
Taguchi	0.0670	(MSE)	[56]
SMGA	1.7220	(MSE)	[57]
APSO	0.0010	(Position)	[58]

**Table 3:** Computational tool performance.

Number of Robots	k-Step	Time (s)	Max Memory (MB)
**10**	1	0.11	246,438
	2	0.17	250,492
	3	0.23	261,342
	4	0.47	267,998
	5	0.51	271,122
**30**	1	0.11	342,120
	2	0.23	358,224
	3	0.43	370,412
	4	1.21	381,102
	5	1.35	403,444
**70**	1	0.13	560,178
	2	0.22	592,174
	3	0.47	611,526
	4	1.32	624,104
	5	2.44	652,666

## Data Availability

Data are contained within the article.

## Abbreviations

ANN	Artificial Neural Network
DCS	Discrete Controller Synthesis
GWO	Gray Wolf Optimization
ACO	Ant Colony Optimization
ABC	Artificial Bee Colony
PSO	Particle Swarm Optimization
COA	Coyote Optimization Algorithm
IK	Inverse Kinematic
MH	Inverse Kinematic
SDCS	Symbolic Discrete Controller Synthesis
DOF	Degree-of-freedom
DH	Denavit–Hartenberg
PID	Proportional Integral Derivative
FOPID	Fractional Order PID
CAD	Computer-Aided Design
MSE	Mean Square Error
APSO	Adaptive Particle Swarm Optimization
